# Genomic landscape of CpG rich elements in human

**DOI:** 10.1186/s12862-016-0864-0

**Published:** 2017-02-07

**Authors:** Vladimir N. Babenko, Irina V. Chadaeva, Yuriy L. Orlov

**Affiliations:** 1 Institute of Cytology and Genetics SB RAS, Lavrentyeva, 10, 630090 Novosibirsk, Russia; 2 Novosibirsk State University, Pirogova, 2, 630090 Novosibirsk, Russia

## Abstract

**Background:**

The studies on CpG islands (CGI) and Alu elements functions, evolution, and distribution in the genome started since the discovery in nineteen eighties (1981, 1986, correspondingly). Their highly skewed genome wide distribution implies the non-random retrotransposition pattern. Besides CGIs in gene promoters, CGIs clusters were observed in the homeobox gene regions and in the macrosatellites, but the whole picture of their distribution specifics was not grasped. Attempts to identify any causative features upon their (genome wide) distribution, such as the DNA context mediated preferred insertion sites of Alu repeats, have been made to ascribe their clusters location.

**Methods:**

Recent emergence of high resolution 3D map of human genome allowed segregating the genome into the large scale chromatin domains of naturally observable nuclear subcompartments, or Topologically Associated Domains (TADs), designated by spatial chromatin distribution. We utilized the chromatin map to elucidate relations between large scale chromatin state and CpG rich elements landscape.

In the course of analysis it was confirmed that genes, Alu and CGI clusters maintain obvious, albeit different in strength, preference for open chromatin. For the first time it was clearly shown that the clusters density of the Alu and CGIs monotonically depend on the chromatin accessibility rate. In particular, the highest density of these elements is found in A1 euchromatin regions characterized by a high density of small length genes replicating in the early S-phase. It implies that these elements mediate (CGIs) or are a side element (Alus) of chromatin accessibility.

**Results:**

We elucidated that both methylated and non-methylated CGIs display the affinity to chromatin accessibility. As a part of comparative genomics section, we elucidated that the dog’s genome non-canonical structure, outstanding in mammals for its high CGIs abundance compared to gene number, is explained by the presence of dense tandem CGI extended hotspots (500 kb on average) in subtelomeric and pericentromeric regions with highly skewed CG content, and not by CGIs global distribution pattern shift.

**Conclusions:**

The study underlines the close association of CG-rich elements distribution with the newly introduced large scale chromatin state map, proposing a refined standpoint on interrelation of aforementioned genome elements and the chromatin state. To our expertise, the TAD-associated partition model employed in the study is likely the most substantial one regarding CpG rich clusters distribution among the whole genome chromatin/isochores maps available.

**Electronic supplementary material:**

The online version of this article (doi:10.1186/s12862-016-0864-0) contains supplementary material, which is available to authorized users.

## Background

The total number of CpG sites in human genome comprises around 28.3 mln instances [1]. That is less than 1% of genome compared with 4.4% expected given 42% GC content due to C- > T mutation shift following the frequent 5′ cytosine deamination in CG doublet [2]. Aside from randomly distributed CpG sites in mammalians, there are two major expansions of them in primates, namely: Alu retrotransposons [3] and CpG cluster units, CGIs [2]. The share of Alu CpGs was estimated up to 30% as early as in 1993 [4] and now is confirmed to be more than 25% [1], while CGIs account for only 2% of CpG content in human [5].

With that, CGIs proved to represent a highly specific marker for open chromatin [2]. In particular, unmethylated CGI and genes association is reported as early as in 1987 [6].

The average CpG content of CGI is 18%, and Alu is about 3.6% (still higher than 1% genome average), which allows considering both of them CpG-rich elements. Three major Alu families differ significantly in CpG content: from 2.5% on average in AluJ, to 3.3% in the most abundant AluS family, and up 6% in the young AluY sequences. Notably, CGIs and Alu complement in methylation pattern: 70% of CGIs are completely unmethylated, while around 70% of Alus are methylated in all tissues/cell types.

The CGIs play an overwhelming role within promoters of about 70% genes. One of current views of the promoter CpG enrichment implications is the alteration of DNA conformation to non B-DNA (Z-DNA) structure and thus reduced nucleosome affinity [5, 7–12]. It was shown that the length of CGI and nucleosome depletion rate significantly correlate: long CGIs are nucleosome free, while small CGIs can attract/position nucleosomes [10]. DXZ4 macrosatellite spanning 45–120 kb [13] and other CGIs in intergenic regions and gene deserts display basal transcription in a majority of cell lines featuring the accessible (open) non-compacted chromatin [13]. This was also confirmed by DHS and CGI co-location, which was also evolutionary conserved [14].

Intriguingly, CpG-rich Alu families tend to distribute their CpG sites at the distance of 31–32 bp, which make them prone to nucleosome binding in mammals [11, 15]. It is worth mentioning that CG dinucleotide signal is the only one among all 16 dinucleotides which manifests the significance detected by autocorrelation analysis of nucleosome positioning in mammals [15]. Also, the CGI mediated nucleosome depletion doesn’t essentially depend on methylation state [11], but that way they become inaccessible to transcription factors binding and protected from spurious RNA PolII complexes assembly. Besides, there were reports of nucleosome dips around polyadenilation sites (PAS) [16, 17].

Analysis of causes of CGIs conservation phenomena during evolution revealed that they evolve in three distinct regimes: a) hypodeaminated (non-methylated promoters); b) methylated with stable CpG content due to Biased Gene Conversion; c) CG-rich exons. There is a small fraction of pseudo-CGIs, which arise due to spurious clustering [18].

The largest CGIs class represents hypomethylated 5′ located CGI promoter regions responsible for PolII based transcription initiation in 70% of genes. Still, there are smaller (comprising around 25% of CGIs total), but nevertheless essential CGI classes within the vicinity of genes that are differentially or constitutively methylated and are presumably involved in tissue and temporal specificity of gene expression regulation. The functional implication of such CGIs is supported by non-random synonymous substitutions inferred from CGI-CDS overlapping instances [19, 20].

Based on previous studies, the number of CGIs and protein coding gene numbers are approximately equal in the majority of mammals and correlate chromosome wise [21–23]. But there is certainly not one to one correspondence of the CGIs and genes: a range of CGI clusters can be observed in gene deserts, e.g. macrosatellites D4Z4 [24, 25], DXZ4 [13] and others. Backwards, there is plenty of tissue specific gene clusters lack of CGIs (e.g. olfactory receptors). For HOX genes, there are multiple CGIs along each gene [20]. Thus, the issue of genes and CGI relation renders further elaboration.

Advances in whole genome epigenetic marks mapping by novel Chip-Seq [26] and Chia-PET [27] techniques have culminated into the elucidation of 3D chromatin conformation. To date it was recognized that the chromatin domains are the ultimate units of chromosome organization [28]. While Chip-seq experiments feaure high resolution maps of 200–400 bp spans usually corresponding to transcription factors and chromatin remodeling binding sites along with the more extent histone modifications areas, the chromatin 3D conformation map maintains large scale domain size of several hundred kbs [28–30]. They were named as Topologically Associated Domains (TADs). The TADs preserve essentially cell-type invariant architecture [31], though, since TADs maintain hierarchical structure within, the cell type specific chromatin conformations also take place on a minor scale [29].

Large scale chromatin domains were primarily segregated into Active domains (A type) of early replication timing chromatin, and lamina associated domains (LAD) (B type) conferring heterochromatic domains with late replication [30, 32]. The replication timing analysis also identified the timing transition regions (TTR) as a separate domain type [32]. At the same year HiC conformation capture analysis study [31] elaborated A and B domains into 6 classess (A1, A2, B1, B2, B3, B4) based on analysis of several epigenetic markers profiles within observable chromatin contact domains related to the similar nuclear subcompartments [31]. The attempts to adequately segregate the domains into the chromatin state variants accounting for multiple factors are ongoing in chromatin conformation studies [28]. Notable obstacles herein are that certain domains change its state in the course of embryonic development and cell speciation [33]. Currently, 3D chromatin conformation research reached the point of making possible to model chromatin architecture based on primary data of classification of loci into chromatin types and a catalog of the positions of chromatin loops [34, 35].

Concerning our study of CpG rich elements and TADS relation, the differential enrichment of A and B TADs with SINEs, such as Alu in human and B1 in mouse, has been reported previously [36]. The extended TADs classes repertoire employed in our study allowed more elaborated quantification of the feature. To our knowledge, no straightforward TADs mediated CGIs content analysis has been made to date.

## Methods

### Genome data sources

We downloaded data set from UCSC genome browser database (genome.ucsc.edu; [37]). We used human genome version hg19; mouse genome version mm10; dog genome version canFam3. For CGIs annotation we used tables named *Cpgislandext* in all cases. Genes were downloaded from the same genome versions, using *refGene* tables for human and mouse, and *ensGene* table for the dog annotation. Transposon locations for human were retrieved from RMSK table (hg19). Genes were defined by the distinct transcription start site.

### Correlation significance

Correlation analysis was performed with Pearson correlation coefficient, its significance was ascertained by using Student *t* test in the form:

$$ \mathbf{t}=\frac{\mathbf{r}\sqrt{\mathbf{n}\mathbf{\hbox{-}}\mathbf{2}}}{\sqrt{\mathbf{1}\mathbf{\hbox{-} }{\mathbf{r}}^{\mathbf{2}}}}, $$

where *r* – Pearson correlation coefficient, *n* – sample size (number of bins). *Df* = *n-1*.

### ANOVA analysis

ANOVA analysis of TADs subcompartment classes for elements density has been performed using XLStat software (www.XLStat.com). To test the deviation significance of the elements density between the TAD subcompartment classes we used Tukey HSD test.

### DNA methylation datasets

Methylation profiles for 63 cell lines and one primary liver cell in ENCODE were downloaded from UCSC genome browser HAIB Methyl450 track [38]. The score of the methylation value associated with each CpG site was defined as the beta value (1) (Illumina’s Bead Studio software with the Methylation Module v3.2) multiplied by 1000.

1 $$ {\beta}_k=\frac{I_k}{{\displaystyle {\sum}_m}{I}_m+100} $$

where *I*_*k*_ – methylation intensity value on the particular CpG site, the sum is genome wide intensity (approximately 450,000 CpGs)

CGI methylation score was calculated as the average of inner CpG sites methylation scores presuming CGI methylation homogeneity [39].

### DNase Hypersensitive Sites (DHS) set

DHSs are ENCODE elements represented by short (100–200 bp) DNA fragments. They indicate open or accessible chromatin where DNA is not tightly wrapped within a nucleosome, leaving the sequence accessible to DNA-binding proteins [40]. DHSs largely correspond to transcription factor binding sites, chromatin remodelers and other DNA binding proteins sites on DNA. In a database, they are supplied with scores corresponding to intensity rate specific for each cell line considered.

We used 2.8 mln DHS sites compiled in [14] from 112 human samples representing 72 cell types, to characterize 100 kb genome bins by DHS density.

## Results

### Distribution features of the elements considered

We used 100 kb bins for the global domain wide analysis of human genome. The distribution basic statistics of Alus, DHSs, CGIs, and genes in 100 kb bins are presented in the Table 1.

**Table 1:** Distribution features of 5 DNA elements considered across 100 kb bins

DNA elements	Avg	Median	Std dev	Min	Max	df
*L1*	32.6	31.0	14.3	1	211	29,209
DHS	101.8	89.0	62.7	1	341	28,380
CGIS	1.8	1.0	1.5	1	21	10,518
Alus	40.9	26.0	35.5	1	235	28,182
Transcripts	2.3	2.0	2.0	1	53	14,303

We ascertained that Alu, transcripts and CGIs feature exponential distribution (see distributions in Additional file 1: Figure S2), which implies significant number of dense clusters [18, 41, 42], while *L1*, DHS maintain binomial distributions ([43]; Additional file 1: Figure S3).

To perform DHS vs other elements comparison we split overall 29,381 100 kb euchromatic bins into 292 clusters ordered by overall DHS number spanning from 0 (26 bins) to 341 (1 bin) DHS entries. The number of DHS per bin was treated as a rough scale chromatin accessibility signature attributable for 100 kb genome segments, and was used as a grouping factor for other DNA elements comparison (Alu, transcripts, CGI distributions).

### Nuclear subcompartments map of genome (Topologically associated domains, TADs)

In 2014 Cell report [31] a high resolution 3D map of human chromatin in a range of cell lines was reconstructed. In the course of analysis the authors delineated 6 major classes of nuclear subcompartments (TADs), which partition whole genome contact domains volume by chromatin state analysis. Six classes comprise two euchromatic (A1, A2) and 4 heterochromatic (B1, B2, B3, B4) subcompartments [31]. Each class is characterized with specific histone modification profiles [31]. Since these are large – scale domains (median length 185 kb; 450 kb on average; [31]) they are compatible with our 100 kb resolution. The overall state of the nuclear subcompartments is presented in Table 2. We excluded B4 class from consideration since it comprised only 25 domains specific for chromosome 19 [31], as well as NA domains.

**Table 2:** Distribution of nuclear subcompartments (TADs) in human genome [31]

	Number	Avg length (kb)	Std dev (kb)	Total length (Mb)
A1	490	818	1217	400.6
A2	1249	465	547	581.4
B1	896	390	427	349.4
B2	504	864	1164	435.7
B3	685	1249	1639	855.5
B4	25	440	495	11
N/A	215	1157	3404	248.7
Total	4064			2882.3

#### A1, A2 – open chromatin, B1-B4 – heterochromatin

According to description in [31], “A” euchromatin segment features enrichment in open chromatin histone marks: H3K36me3, H3K79me2, H3K27ac, and H3K4me1. A1 chromatin state completes replication at early S phase, while A2 proceeds replicating up to the mid – S phase. A2 is enriched with H3K9me3 and contains longer genes. Subcompartment B1 is enriched with H3K27me3 and depleted of H3K36me3 marks, representing facultative heterochromatin. Subcompartment B2 includes the majority of pericentromeric heterochromatin and is enriched at the nuclear lamina and at Nucleolar Associated Domains (NAD). Subcompartment B3 is enriched at the nuclear lamina and is depleted at NADs, thus corresponding to constitutive heterochromatin. B4 comprises a range of marks representing highly ambiguous chromatin pattern. It features strong enrichment for both activating chromatin marks, such as H3K36me3, H3K4me3, H3K27ac, and heterochromatin-associated marks, such as H3K9me3 and H4K20me3. B4 contains 130 of the 278 KRAB-ZNF genes in the genome which is highly non-random [31]. More details on the subject could be ascertained from the original work [31].

### CGIs association with genes

The chromosome wise dependence of CGIs and genes was reported previously for a range of species genomes, including dog genome featured with highly skewed CGI to gene ratio due to abundance of CGIs [22]. We replicated these results for three mammalian species (Additional file 1: Figure S1; *P* < 1.3E-25 for human, *P* < 3.5E-30 for mouse and *P* < 1.7E-31 for dog). This correlation could apparently be expected as 60-70% of CGIs overlap promoters [44] but we aimed to assess it more explicitly.

We first approached the genes-CGI relations by considering 100 kb bins genome wide. The vast number of genome 100 kb bins lack both CGIs and genes. To assess this, we calculated 2x2 contingency tables for human, mouse, and dog, correspondingly (Table 3). One may see that non-randomness of joint gene/CGI deserts as well as their co-occurrence is highly significant (Table 1). In particular, the concordant classes for human (no genes and CGIs and at least 1 gene and at least 1 CGI) comprise 76% of bins leaving only 24% of discordant bins (Table 3). This joint distribution leaves no doubt of overall interrelation of CGIs and genes locations.

**Table 3:** 2×2 contingency tables of 100 kb bins distribution for human, mouse, and dog

	Human		Mouse		Dog	
	No genes	At least 1 gene	No genes	At least 1 gene	No genes	At least 1 gene
no CGIs	12,684	4759	13,276	4910	11,309	3454
at least 1 CGI	2267	9544	1259	7184	3361	8896
	*χ* ^2^ = 8071		*χ* ^2^ = 7848		*χ* ^2^ = 6528	

df = 1 *P* < 1E-306* for all Pearson 2×2 table chi-square tests

*P value was calculated for *χ* ^2^ = 1200 only due to the floating point accuracy limit (32 bits word size) for values greater than that

We further extended 2x2 tables analysis and built up the distributions of CGIs and genes based on their density across 3 species. The CGI vs gene numbers density per 100 kb distributions were highly correlated with approximately the same rate as chromosome wise (Additional file 1: Figure S2; *P* < 1.6E-31; df = 20 for human; *P* < 1.4E-23; df = 15 for mouse and *P* < 3.6E-30; df = 18 for dog). Notably, CGIs and genes correlate also in dog genome irrespective of nearly 2-fold excess of CGIs number over genes number in this species [22]. Thus, the co-variation of CGIs and genes densities across 100 kb segments in mammalian genomes is significant on a coarse grain even given the multiple (but not predominant) spurious independent clusters (Additional file 1: Figure S2).

Further we proceeded with human genome only in elucidating other features in elements distribution due to the lack of appropriate data for other species.

### Open chromatin and elements density

Next we assessed if CGIs vs genes co-clustering may be mediated by chromatin accessibility factor. To check that, we built up the joint distributions of DHSs density against CGIs and genes (Additional file 1: Figure S3, S4, S5). The regression plots of the elements against 292 bins of DHS density rate (see materials and methods) revealed that the correlation of the DHS and genes number per DHS bin is *r* = 0.92; *P* < 1.4E-120 (Additional file 1: Figure S3a). The correlation between DHS and CGIs was the highest due to the inherent features of CGIs: *r* = 0.97; *P* < 1.1E-188 (Additional file 1: Figure S5a). So, the chromatin accessibility is an ultimate factor for clustering of CGIs and genes (Additional file 1: Figure S7).

To confirm it in another way, we employed large scale whole genome chromatin state segmentation map from [31] to assess the distribution of CGIs and genes across different chromatin states.

**Fig. 1:**
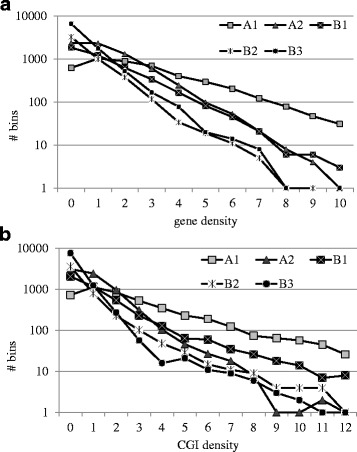
Breakdown of gene (**a**) and CGI (**b**) densities into five chromatin classes [31]

As can be seen from Fig. 1, the zero and one gene and CGI bins consist largely of B2- B3 heterochromatin, and also of A2 euchromatin type. As long as gene density increases, the major gene and CGI clusters containing class confines to A1 open chromatin (Fig. 1).

**Fig. 2:**
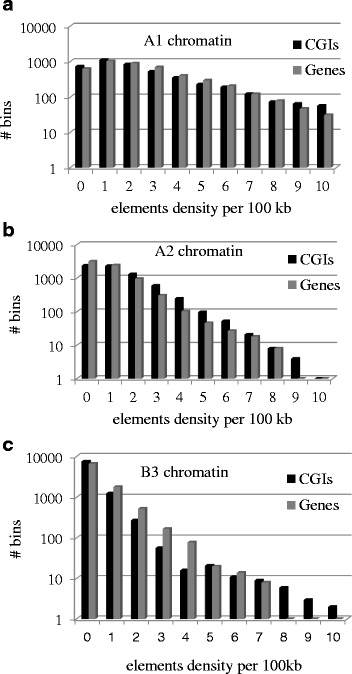
Distribution of open chromatin (A1, A2) and heterochromatin (B3) in CGIs and genes. The correlations are: **a** *r* = 0.98 t = 19.3; df = 9 *P* < 6.1E-9; **b** *r* = 0.97; t = 18.4, df = 9; *P* < 9.2 E-9; **c** *r* = 0.99, t = 38.0, df = 9, *P* < 1.5E-11

We compared the distribution of CGIs and genes across 3 largest classes of chromatin (Fig. 2). We observed the gene dense open chromatin of A1 type preference for both CGIs and genes, while A2 and B3 chromatin classes distributions decay rapidly with increasing elements density. High correlation between CGIs and genes are observed (*P* < 1E-9 for all three cases).

### Methylation state and chromatin accessibility

Based on average methylation state computed across 63 HAIB Methyl cell lines data, we found that approximately 20% of CGI (4057) exceeded empirically chosen threshold: average methylation score >400. They were assigned as “(hyper)methylated” states, and “hypomethylated” ones otherwise. Due to the U-shaped methylation score distribution across the data we unintentionally included a range of differentially methylated CGIs into “hypermethylated” sample, but they are not abundant.

Chromosome wise analysis revealed high correlation of genes number with both unmethylated and methylated CGI clusters (Additional file 1: Figure S11). Next we plotted the total, hypomethylated and hypermethylated samples against DHS densities (Additional file 1: Figure S5). To assess the significance of correlation between the CGIs number and DHS densities, we performed regression analysis of the corresponding plots (Additional file 1: Figure S6). We assessed the correlation significance as *r* = 0.97, *P* < 1.1E-188 for total CGIs set; *r* = 0.96, *P* < 1E-169 for hypomethylated CGIs and *r* = 0.95, *P* < 1.7E-154 for hypermethylated CGIs sample. We also observe twice less DHS overall density in hypermethylated set compared to hypomethylated ones probably due to the observed 4-fold smaller size of hypermethylated vs hypomethylated CGIs sets.

These results indicate that there is no straightforward correlation between CGI methylation and binding sites repulsion, which is compliant to current observations in [45, 46]. It is plausible that though not all transcription factors are blocked from binding to CGIs by methylation, still the chromatin status could be altered [45].

### Alu sequences and chromatin content

While the CGIs and genes exhibit highly specific exponential distribution, Alu sequences are a lot more abundant and, to our knowledge, no global highly specific discriminating factor for their clustering was reported. In particular, 42% of Alu sequences reside within gene loci, which is very close to random insertion pattern since genes occupy about 40% of genome [42]. Chromosome wide Alu distribution closely follows chromosome length, also implying random nature of Alu distribution independent of genes and CGI clusters, at least chromosome wide. Still, there are Alu clusters that were reported abundant genome wide and were linked to various factors including recombination rate and others [41].

We analyzed DHS and Alu distributions (Additional file 1: Figure S3b, S4b, S8a). It underlies that, in contrast with CGIs/genes, the noticeable number of Alu clusters reside in DHS – poor regions that, in accordance with previous randomly based chromosome wise entities, point to a largely stochastic nature of Alu insertions and/or probably other factors besides the open chromatin state, involved in Alu insertion preference and cluster expansions.

Nevertheless, Additional file 1: Figure S8a underscores the overall trend of Alu clusters to open chromatin, which is consistent, but with small statistical significance due to a big standard variation and small slope of regression line. Our interconnection link of this TE with CGIs and genes is thus rather speculative, based on the specific trends of particular subfamilies vs the chromatin state.

**Fig. 3:**
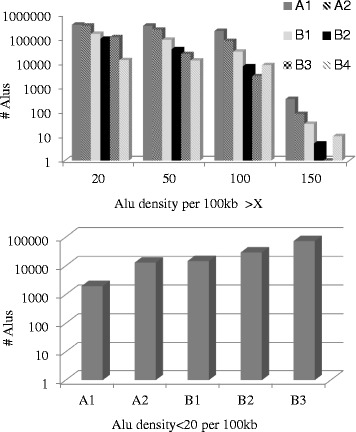
Dependence of Alu density ‘tails’ on chromatin class

We segregated Alu density 100 kb bins by chromatin type (Fig. 3), observing their distinct open chromatin preference with clustering (Fig. 3, Additional file 1: Figure S9a). No Alu clusters with density >150 per 100 kb was observed in the most abundant B3 heterochromatin (Fig. 3).

The overall statistic of Alu content in chromatin classes (Alu overall densities per 100 kb total averages) is: A1(66), A2(42), B1(44), B2(34), B3(30), B4(110). Omitting B4 class spanning only 10 Mb total, A1 is manifested as the preferred chromatin class for Alu insertion.

When we applied the chromatin breakdown procedure for Alu subfamilies, we observed that AluY clusters prefer the gene related A1 chromatin, while AluJ and AluS clusters partially reside in open chromatin (A1, A2) with no specific preference (Additional file 1: Figure S10).

We also employed *L1* retrotransposons analysis to assess chromatin distribution. We found that *L1* retrotransposons are randomly distributed in the genome except for highly DHS dense regions (Additional file 1: Figures S3c and S4c), and, consequently, gene dense A1 segments (Additional file 1: Figures S8b and S9b). Considering long *L1* clusters, they distinctly reside in constitutive heterochromatin lack of DHS (Additional file 1: Figure S8c). Thus, Alus and *L1* retrotransposons partially complement each other both in mode of distribution and in chromatin preference.

### ANOVA analysis of the genes, CGIs, and retrotransposons distribution across TADs

ANOVA analysis was employed to complement the regression analysis results. We considered elements variation across 4064 TADs (Table 2) to see the overall elements density.

**Fig. 4:**
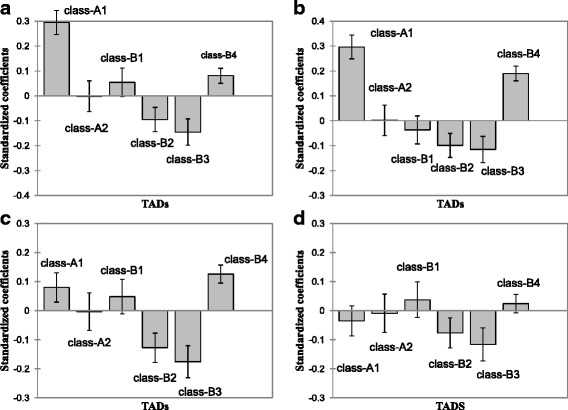
ANOVA results on CGIs (**a**), small genes (length < 20 kb) (**b**), Alu (**c**) and *L1* (**d**) densities across TAD classes

From Fig. 4 and ANOVA tests we may make 3 essential conclusions concerning TADs content of 4 elements:

- B2-B3 heterochromatic domains are in a deficit of all elements considered and form a joint group for all 4 elements based on Tukey HSD test (*P* < 0.5; <1; <0.77; <0.82 for (a), (b), (c) and (d), correspondingly);
- B4 domains are enriched in all of the elements. B4 groups with A1 chromatin in (a) (*P* < 0.994) and (d) (*P* < 0.34) cases, according to Tukey HSD test.
- A2 chromatin maintains genomic average in all of the cases.

Based on the Tukey HSD test, no dramatic differences were observed between chromatin states for *L1* distribution (Fig. 4c), confirming its random retrotransposition and broad variation in density in all classes. Still, when considering long *L1,* which comprise only 10% of the total pool of *L1* genome instances, the situation changes sharply in favor of B2/B3 preference (Additional file 1: Figure S8c).

A1 and A2 TADs significantly differ in all cases except (d), which makes the point of quite distinct patterns of euchromatin in human genome given both of them maintain open chromatin signature in histone and other marks [31]. Notably, while B4 maintains reported repressive histone marks [31], and in our case it comprises significant number of *L1* transposable elements [47], still, many of its features are also similar to gene dense A1 chromatin (Fig. 4a-c), pointing at its reported duality [31, 47].

### CG rich elements and clustering properties

**Fig. 5:**
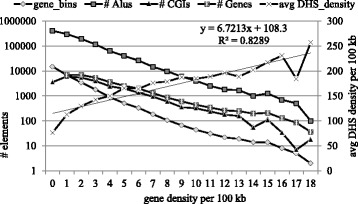
Distribution of CGIs and Alus in gene-defined bins categories compared to average DHS density. The correlations between the subjects (without 0 – class) are: Alu-Genes: (*r* = 0.93, t = 10.54; df = 17; *P* < 2.3E-10); Alu-CGIs (*r* = 0.96 t = 16; df = 20; *P* < 5.0E-14); Genes-CGI (*r* = 0.98, t = 21.2; *P* < 2.4E-21). The average DHS density linear regression presented on the plot is also significant: *r* = 0.91; t = 10.12; df = 18; *P* < 1.1E-8)

We selected gene density distribution bins to plot CGIs and Alu clusters distribution against (Fig. 5). Indeed, we observed similar distribution of gene densities in all 3 elements (Alu-Genes correlation: *r* = 0.93, *P* < 2.3E-10) Alu-CGIs (*r* = 0.9, 6 *P* < 5.0E-14); Genes-CGI (*r* = 0.98; *P* < 2.4E-21)). In addition, there is a significant linear trend of DHS density (*P* < 1.1E-8) with gene density increase. All that reflects the notion that gene dense regions are abundant with CGIs and Alus and feature open chromatin.

## Discussion

The study aims at gaining the insight on the distribution specifics of several DNA elements, specifically genes, CGIs and Alus, from the large-scale chromatin landscape standpoint. We found that chromatin accessibility rate is the major factor of joint clustering of these elements. Heterochromatic genomic segments spanning nearly a half of the human genome are void of both CGIs and genes, making their distribution highly skewed genome wide. This keeps valid for others species such as mouse and dog (Table 3, Additional file 1: Figures S1 and S2) and might be extended on other mammals.

With the help of coarse genome partition by 100 kb non-overlapping segments we showed that CGIs extremely closely associate with the genes. It was shown that this association is consistent and independent of the proportion of the CGIs vs genes number, which is approximately equal in the mammals majority. In this regard, for the dog genome, characterized by an unusual twofold excess of CGIs instances over the genes number, it was found that an excess can be explained by the relatively few instances of CGI extended clusters. At the same time the proportion and localization of genes and the vast majority of CGIs in the dog genome are coordinated as in other mammals (Additional file 1: Figure S2c).

Indeed, when we mapped the dense CGI clusters onto the dog genome, we clearly observed their highly non random grouping at subtelomeric and pericentromeric regions sparse of genes (Additional file 1: Figure S12). These specific telomeric and centromeric superclusters spanning 500 kb on average were observed at virtually all dog chromosomes (subtelomeric regions in particular) and comprise at least 30% of the annotated CGIs (16,000 instances by rough approximation). The subtelomeric CGI clusters have been observed in cat, horse, and bovine, as well as occasionally in human and have been reported in previous studies [21], but not with the same expansion rate and abundance as observed in dog. The apparent function of these CGI clusters is not currently elucidated, but may be related to the repeated nature of the DNA, characteristic for these regions.

Employment of the expanded chromatin spectrum using chromatin signatures inferred from topological associated domains [31] revealed that the distributions of CGIs and genes are highly similar chromatin wise both on the fine, DHS-mediated resolution (Additional file 1: Figures S3–S6) as well as on the coarse-grained topological chromatin domains (Figs. 1 and 2). This makes us suggest that CGIs are the inherent elements of the genes irrespective of their methylation state and location. The independent tandem expansions of CGIs and gene families do not affect the total trend due to their minority.

ANOVA analysis of the elements content in 6 TAD subcompartments (Fig. 4) corroborated the inferences derived from regression analysis. Additionally, TAD classes interrelations could be observed for the distinct elements content underlining the specific features of TAD classes.

It was elucidated that the genes, Alu and CGIs clusters density is monotonically dependent on the chromatin state derived by DHS densities (Additional file 1: Figures S7 and 8). Considering the TADs mediated chromatin partition, the highest density of these elements is found in A1 euchromatin (Additional file 1: Figures S3–S6 and S10). These regions are characterized by a high density of small length genes, and replicating in the early S-phase (G1, S1 phases) [31]. Thus, these elements mediate or are a side element of chromatin accessibility. It is likely that Alus use open chromatin, but CGIs create it.

Using L1 transposable elements as a background we showed by ANOVA (Fig. 4) and distribution analysis (Additional file 1: Figures S8 and S9) that L1 instances are distributed independently of chromatin state, while subset of long L1 sequences are distinctly resided in heterochromatin.

In this context we’d like to draw attention to chromosome 19 due to the extraordinary CG-rich elements density, both for CGIs (Additional file 1: Figures S1a and S11) and Alus (98 Alus per 100 kb; next highest is chr17 with 70 per 100 kb, and genome average is 40 Alus per 100 kb; *P* < 0.004 by ESD test). Given it is more than twofold dense in gene number than any other chromosome [48], it thus implicitly confirms the close coordination of genes and CG-rich elements. Importantly, chromosome 19 also is the most hypermethylated one chromosome wide (Additional file 1: Figure S11b), while hypomethylated at fetal stage [49] (Additional file 1: Table S1), which may imply specific mode of evolution of this particular chromosome by gene duplications [48, 50], and the abundance of development genes [47]. In particular, it was proposed that the random promoter methylation within the newly emerged paralogous gene pair in the course of embryonic reprogramming stage may highly increase the chance for both genes to keep the functionality upon duplication [50].

## Conclusions

One of the possible reasons of chromosome 19 observed evolutionary mode may be that it comprises more than a hundred of KRAB-ZNF genes organized in clusters and represents “defense” system against hypomethylated transposable elements in embryogenesis. It is responsible for identification of non-methylated CpG elements located at retrotransposons. Upon recognition they recruit TET protein, which, in turn, recruits heterochromatin modifiers to repress them, and subsequently change methylation status [51, 52]. These particular clusters reside in chromosome specific B4-heterochromatin type nuclear compartments [31] which demonstrate even higher than A1 abundance with Alu clusters (Fig. 4). It may imply B4 accessibility in early embryogenesis, and thus a high fixation rate. The latter hypothesis is also supported by B4 non-random abundance with other transposable elements clusters, such as *L1*M3a [49] and others.

Coherent with CpG abundance, chromosome 19 contains single A1 euchromatin class, and virtually no constitutive heterochromatin B3 and euchromatin A2 domains, manifested as the largest chromatin classes occupying 50% of the genome (Table 2). The A1 “style” of gene arrangement on chromosome 19 underlies the fact that the genes located within this chromosome maintain the average length shorter than the genome wide average with a high statistical significance.

Notably, A1 chromatin state relates to early replicated domains [31] and, hence, comprises multiple origin of replication sites (ORI) that fire shortly after G phase. As long as there could be some analogy with the insertion pattern of P-elements in *D.melanogaster*, where ORI sites are shown to be the hotspots of P-element insertions [52], we speculate that Alu retrotransposons may display the same affinity to ORI sites attributable to open A1 chromatin, and, hence populate the ORI-related chromatin.

Large scale features of open chromatin may affect the gene context at a lower resolution level. Besides well known CGIs preference to 5′ gene regions, we observed reported previously high density of CGIs in 3′ gene regions [17, 22]. Significant portion of Alus reside both in promoter regions [53] and in 3′ UTR elements [54]. Given both transcription start and end sites maintain specific epigenetic markers [26], we put forward the hypothesis that both of these elements form the gene locus architecture accommodating gene expression maintenance. In particular, CGIs and Alu may cooperate in promoter regions given the recent observation that highly expressed housekeeping genes maintain their CpG-promoter flanked with hypermethylated “shells”, possibly accommodating more accurate RNA PolII positioning [55] and involving methylated Alu retrotransposons as CGI “scaffolding” factor [53].

The non-random distribution of the elements considered mediated by chromatin state may be practically utilized by applications in the association studies for refining their statistical significance in location/clustering by incorporating prior knowledge on the particular chromatin state in a model.

The evolutionary implications of the elements within chromatin state context may confer that *L1* and CGIs clusters could lead to the chromatin state setting in the course of expansion, while Alu elements seem rather opportunistic in location preference due to non-autonomous nature.

Finally, the observed CGIs expansion dynamics may impact the issue of the genome CpG content maintenance. CpG methylation rate was recently proposed to be the driving force of genome size evolution within a tradeoff in methyltransferases methylation rate vs genome size [56]. As an example, D. melanogaster and C. elegance minimal genomes phenomena could be explained by the absence of methyltransferases, and, as a consequence, CpG content equaling to the expected one. Thus, it looks like CGI duplication mechanism is of evolutionary importance for maintaining sufficient CpG number. In this regard, some subtle mechanisms apparently ‘compel’ the dog genome to amplify CG-rich tandems within subtelomeric/pericentromeric regions chromosome wide, even given the significantly reduced gene repertoire and corresponding CGI promoters [22], this way aligning CpG (or just GC) content with that of other mammals.

## Additional file

Additional file 1: Supplementary figures. (DOCX 186 kb)

## References

[1] Luo Y, Lu X, Xie H. Dynamic *Alu* methylation during normal development, aging, and tumorigenesis. Biomed Res Int. 2014:784706. http://dx.doi.org/10.1155/2014/784706. 10.1155/2014/784706 PMC4163490 25243180

[2] Bird AP (1986). CpG-rich islands and the function of DNA methylation. Nature.

[3] Rinehart FP, Ritch TG, Deininger PL, Schmid CW (1981). Renaturation rate studies of a single family of interspersed repeated sequences in human deoxyribonucleic acid. Biochemistry.

[4] Hellmann-Blumberg U, Hintz MF, Gatewood JM, Schmid CW (1993). Developmental differences in methylation of human *Alu* repeats. Mol Cell Biol.

[5] Deaton AM, Bird A (2011). CpG islands and the regulation of transcription. Genes Dev.

[6] Bird AP (1987). CpG islands as gene markers in the vertebrate nucleus Trend. Genetics.

[7] Schones DE, Cui K, Cuddapah S, Roh TY, Barski A (2008). Dynamic regulation of nucleosome positioning in the human genome. Cell.

[8] Ramirez-Carrozzi VR, Braas D, Bhatt DM, Cheng CS, Hong C (2009). A unifying model for the selective regulation of inducible transcription by CpG islands and nucleosome remodeling. Cell.

[9] Choi JK (2010). Contrasting chromatin organization of CpG islands and exons in the human genome. Genome Biol.

[10] Fenouil R, Cauchy P, Koch F, Descostes N, Cabeza JZ (2012). CpG islands and GC content dictate nucleosome depletion in a transcription-independent manner at mammalian promoters. Genome Res.

[11] Jimenez-Useche I, Ke J, Tian Y (2013). DNA methylation regulated nucleosome dynamics. Sci Rep.

[12] Jonkers I, Kwak H, Lis JT (2014). Genome-wide dynamics of Pol II elongation and its interplay with promoter proximal pausing, chromatin, and exons. Elife.

[13] Horakova AH, Moseley SC, McLaughlin CR, Tremblay DC, Chadwick BP (2012). The macrosatellite DXZ4 mediates CTCF-dependent long-range intrachromosomal interactions on the human inactive X chromosome. Hum Mol Genet.

[14] Sheffield NC, Thurman RE, Song L, Safi A, Stamatoyannopoulos JA (2013). Patterns of regulatory activity across diverse human cell types predict tissue identity, transcription factor binding, and long-range interactions. Genome Res.

[15] Bettecken T, Frenkel ZM, Trifonov EN (2011). Human nucleosomes: special role of CG dinucleotides and *Alu*-nucleosomes. BMC Genomics.

[16] Spies N, Nielsen CB, Padgett RA, Burge CB (2009). Biased chromatin signatures around polyadenylation sites and exons. Mol Cell.

[17] Lee CY, Chen L (2013). Alternative polyadenylation sites reveal distinct chromatin accessibility and histone modification in human cell lines. Bioinformatics.

[18] Cohen NM, Kenigsberg E, Tanay A (2011). Primate CpG islands are maintained byheterogeneous evolutionary regimes involving minimal selection. Cell.

[19] Medvedeva YA, Fridman MV, Oparina NJ, Malko DB, Ermakova EO (2010). Intergenic, gene terminal, and intragenic CpG islands in the human genome. BMC Genomics.

[20] Branciamore S, Chen ZX, Riggs AD, Rodin SN (2010). CpG island clusters and pro-epigenetic selection for CpGs in protein-coding exons of HOX and other transcription factors. Proc Natl Acad Sci U S A.

[21] Han L, Su B, Li WH, Zhao Z (2008). CpG island density and its correlations with genomic features in mammalian genomes. Genome Biol.

[22] Han L, Zhao Z (2009). Contrast features of CpG islands in the promoter and other regions in the dog genome. Genomics.

[23] Tsiagkas G, Nikolaou C, Almirantis Y (2014). Orphan and gene related CpG Islands follow power-law-like distributions in several genomes: evidence of function-related and taxonomy-related modes of distribution. Comput Biol Chem.

[24] Zhang YZ, Sun SC, Wu HC, Fan QS, Song YJ (2005). Polymorphism of the D4Z4 locus associated with facioscapulohumeral muscular dystrophy 1A in Shanghai population. Zhonghua Yi Xue Yi Chuan Xue Za Zhi.

[25] Ottaviani A, Schluth-Bolard C, Gilson E, Magdinier F (2010). D4Z4 as a prototype of CTCF and lamins-dependent insulator in human cells. Nucleus.

[26] ENCODE Project Consortium (2012). An integrated encyclopedia of DNA elements in the human genome. Nature.

[27] Li G, Ruan X, Auerbach RK, Sandhu KS, Zheng M (2012). Extensive promoter-centered chromatin interactions provide a topological basis for transcription regulation. Cell.

[28] Rowley MJ, Corces VG (2016). The three-dimensional genome: principles and roles of long-distance interactions. Curr Opin Cell Biol.

[29] Cubeñas-Potts C, Corces VG (2015). Topologically associating domains: an invariant framework or a dynamic scaffold?. Nucleus.

[30] Dixon JR, Gorkin DU, Ren B (2016). Chromatin domains: the unit of chromosome organization. Mol Cell.

[31] Rao SS, Huntley MH, Durand NC, Stamenova EK, Bochkov ID (2014). A 3D map of the human genome at kilobase resolution reveals principles of chromatin looping. Cell.

[32] Pope BD, Ryba T, Dileep V, Yue F, Wu W (2014). Topologically associating domains are stable units of replication-timing regulation. Nature.

[33] Beagan JA, Gilgenast TG, Kim J, Plona Z, Norton HK, Hu G (2016). Local genome topology can exhibit an incompletely rewired 3D-folding state during somatic cell reprogramming. Cell Stem Cell.

[34] Di Pierro M, Zhang B, Aiden EL, Wolynes PG, Onuchic JN. Transferable model for chromosome architecture. Proc Natl Acad Sci U S A. 2016;113(43):12168–12173. doi:10.1073/pnas.1613607113. 10.1073/pnas.1613607113 PMC5087044 27688758

[35] Xu C, Corces VG (2016). Towards a predictive model of chromatin 3D organization. Semin Cell Dev Biol.

[36] Ashida H, Asai K, Hamada M (2012). Shape-based alignment of genomic landscapes in multi-scale resolution. Nucleic Acids Res.

[37] Karolchik D, Barber GP, Casper J, Clawson H, Cline MS (2014). The UCSC genome browser database: 2014 update. Nucleic Acids Research..

[38] http://ucscbrowser.genap.ca/cgi-bin/hgTrackUi?db=hg19&g=wgEncodeHaibMethyl450. (Access date 22 Nov 2016).

[39] Hackenberg M, Barturen G, Carpena P, Luque-Escamilla PL, Previti C (2010). Prediction of CpG-island function: CpG clustering vs sliding-window methods. BMC Genomics.

[40] Wu C (1980). The 5′ ends of Drosophila heat shock genes in chromatin are hypersensitive to DNase I. Nature.

[41] Jurka J, Kohany O, Pavlicek A, Kapitonov VV, Jurka MV (2004). Duplication, coclustering, and selection of human Alu retrotransposons. Proc Natl Acad Sci U S A.

[42] Lander ES, Linton LM, Birren B, Nusbaum C, Zody MC (2001). Initial sequencing and analysis of the human genome. Nature.

[43] Crawford GE, Holt IE, Whittle J, Webb BD, Tai D (2006). Genome-wide mapping of DNase hypersensitive sites using massively parallel signature sequencing (MPSS). Genome Res.

[44] Illingworth RS, Bird AP (2009). CpG Islands --‘a rough guide’. FEBS Lett.

[45] Medvedeva YA, Khamis AM, Kulakovskiy IV, Ba-Alawi W, Bhuyan MS (2014). FANTOM consortium. Effects of cytosine methylation on transcription factor binding sites. BMC Genomics.

[46] Spruijt CG, Vermeulen M (2014). DNA methylation: old dog, new tricks?. Nat Struct Mol Biol.

[47] Vogel MJ, Guelen L, de Wit E, Peric-Hupkes D, Lodén M (2006). Human heterochromatin proteins form large domains containing KRAB-ZNF genes. Genome Res.

[48] Grimwood J, Gordon LA, Olsen A, Terry A, Schmutz J (2004). The DNA sequence and biology of human chromosome 19. Nature.

[49] Spiers H, Hannon E, Schalkwyk LC, Smith R, Wong CC (2015). Methylomic trajectories across human fetal brain development. Genome Res.

[50] Branciamore S, Rodin AS, Riggs AD, Rodin SN (2014). Enhanced evolution by stochastically variable modification of epigenetic marks in the early embryo. Proc Natl Acad Sci U S A.

[51] Long HK, Blackledge NP, Klose RJ (2013). ZF-CxxC domain-containing proteins, CpG islands and the chromatin connection. Biochem Soc Trans.

[52] Imbeault M, Trono D (2014). As time goes by: KRABs evolve to KAP endogenous retroelements. Dev Cell.

[53] Tomilin NV (2008). Regulation of mammalian gene expression by retroelements and non-coding tandem repeats. Bioessays.

[54] Jordan KW, Craver KL, Magwire MM, Cubilla CE, Mackay TFC (2012). Genome-wide association for sensitivity to chronic oxidative stress in *Drosophila melanogaster*. PLoS One.

[55] Varshney D, Vavrova-Anderson J, Oler AJ, Cowling VH, Cairns BR (2015). SINE transcription by RNA polymerase III is suppressed by histone methylation but not by DNA methylation. Nat Commun.

[56] Lechner M, Marz M, Ihling C, Sinz A, Stadler PF (2013). The correlation of genome size and DNA methylation rate in metazoans. Theory Biosci.

