# Supplementary material for: Genomic landscape of CpG rich elements in human
Source: BMC Evol Biol. 2017 Feb 7;17(Suppl 1):19. doi: 10.1186/s12862-016-0864-0 (PMC5333175; doi:10.1186/s12862-016-0864-0)
Supplement: Additional file 1: — Supplementary figures. (DOCX 186 kb) [file 12862_2016_864_MOESM1_ESM.docx]

# Additional file 1 . Supplementary figures. Babenko et al. Genomic landscape of CpG rich elements in human

b

c

Figure S1. Regression of CGIs vs genes chromosome wise in human (a), mouse (b) and dog (c). (a) r=0.96, df=22; P<1.3E-25; (b)r=0.99, df=20; P<3.5E-30 (c) r=0.90; df=38; P<1.7E-31.

Figure S2. Distribution of genes and CGIs densities in the 100 kb bins for 3 species: (a) human, (b) mouse and (c) dog. Correlations between corresponding CGIs and genes distributions are: (a) r=0.99, t=137.3, df=20; P<1.6E-31; (b) r=0.99, t=111, df=15, P<1.4E-23; (c) r=0.99, t=160, df=18, P<3.6E-30.

Figure S3. Distribution of genes (a), Alu (b), and L1 (c) against DHS. 292 DHS bins are considered.

Figure S4. Regression plots for genes (a), Alu (b), and L1 (c) retrotransposons against DHS bins (292 points). Each plot is based on corresponding data in Figure S3. Pearson correlation rates are : genes vs DHS (r=0.92; t=40.2 df=288; P<1.4E-120) alus vs DHS (r=0.88; t=32.2; df=291, P<8.5E-98), L1 vs DHS (r=0.3;t=15, df=290; P<1E-6).

Figure S5. Distribution of CGIs total (a), hypomethylated (b) and hypermethylated (c) against DHS. 289 DHS bins were considered, only those containing CGIs are employed (282- hypermethylated CGIs; 287 – hypomethylated CGIs; 289 – total CGIs).

a

b

c

Figure S6. Regression plots for CGIs total (a), hypo- (b) and hypermethylated (c) against DHS bins. (Data corresponds to Figure S5). Regression significance values are: r=0.97, t=118, df=288, P<1.1E-188 for (a); r=0.96; t=65, df=286, P<1E-169 for (b); r=0.95, t=54, df=281; P<1.7E-154.

b

Figure S7. DHS average density against gene (a) and CGI (b) density. Bracketed is standard deviation of DHS average across corresponding bins.

a

b

c

Figure S8. DHS average density against Alu (a), total L1 (b) and long L1 (c) density. Bracketed is standard deviation of DHS average across corresponding bins.

a

b

Figure S9. Distribution of transposable elements Alu (a) and L1 (b) against nuclear subcompartment chromatin classes. A1, A2 – open chromatin; B1, B2, B3 – heterochromatin (Rao et al., 2014). B4 (HP1-like ) heterochromatin (not shown) comprises plenty of L1 and Alu as well (Vogel et al., 2006).

b

a

c

Figure S10. Distribution of Alu subfamilies AluY (a), AluS (b) and AluJ (c) against nuclear compartment chromatin classes. A1, A2 – open chromatin; B3 – heterochromatin (Rao et al., 2014).

a

b

Figure S11. Regression of CGIs vs genes chromosome wise in human for hypomethylated (a), and hypermethylated (b) CGIs. Total regression is presented at Figure S1. Correlation significances are: (a) r=0.99; df=22; P<2.6E-31; (b) r=0.92 df=22; P<3.1E-22.

Figure S12. Distribution of dense CGI clusters (more than 10 CGIs per 100kb) along the chromosomes (100kb scale) **in dog genome**. We picked the chromosomes with more than 700 CGIs only (except for X chromosome).

**Abbreviations:**

**CGI** – CpG islands;

**DHS** – DNase Hypersensitive Regions (Sheffield et al., 2013).

**References**

1. Rao SS, Huntley MH, Durand NC, Stamenova EK, Bochkov ID et al. (2014) A 3D map of the human genome at kilobase resolution reveals principles of chromatin looping. Cell. 159(7): 1665-1680. doi: 10.1016/j.cell.2014.11.021.
2. Vogel MJ, Guelen L, de Wit E, Peric-Hupkes D, Lodén M et al. (2006 ) Human heterochromatin proteins form large domains containing KRAB-ZNF genes. Genome Res. Dec;16(12):1493-1504.
3. Sheffield NC, Thurman RE, Song L, Safi A, Stamatoyannopoulos JA, et al. (2013) Patterns of regulatory activity across diverse human cell types predict tissue identity, transcription factor binding, and long-range interactions. Genome Res. May; 23(5): 777-788. doi:10.1101/gr.152140.112.
